# SYKT Alleviates Doxorubicin-Induced Cardiotoxicity via Modulating ROS-Mediated p53 and MAPK Signal Pathways

**DOI:** 10.1155/2018/2581031

**Published:** 2018-08-26

**Authors:** Ting Chen, Zhiyong Deng, Ruilian Zhao, Hongmei Shen, Wenhui Li

**Affiliations:** ^1^Department of Nuclear Medicine, Tumor Hospital of Yunnan Province, The Third Affiliated Hospital of Kunming Medical College, Kunming, China; ^2^Department of Combination of Chinese Traditional and Western Medicine, Tumor Hospital of Yunnan Province, The Third Affiliated Hospital of Kunming Medical College, Kunming, China; ^3^Department of Radiotherapy, Tumor Hospital of Yunnan Province, The Third Affiliated Hospital of Kunming Medical College, Kunming, China

## Abstract

*Backgrounds. *Doxorubicin (DOX) is an effective therapeutic drug for malignant tumors; however, its clinical applications were limited by its side effects, especially the cardiotoxicity caused by ROS-mediated p53 and MAPK signal pathways' activation-induced cell apoptosis. Sanyang Xuedai mixture (SYKT) has been reported as an antioxidant agent and attenuated DOX-induced cardiotoxicity by targeting ROS-mediated apoptosis, but the mechanisms are still not fully delineated.* Objective. *This study aimed at investigating whether SYKT alleviated DOX-induced cardiotoxicity by inhibiting ROS-mediated apoptosis and elucidating the role of ROS-mediated p53 and MAPK signal pathways' activation in this process.* Materials and Methods. *Identification, separation, and culture of mouse primary cardiomyocytes. Cells were treated with DOX (1 *μ*M), SYKT (30 mg/mL), or SYKT coupled with DOX. The p53 inhibitor Pifithrin-*α* (PFT-*α*), p38/MAPK inhibitor SB203583 (SB), and JNK inhibitor SP600125 (SP) were used as positive control. Western blot was employed to detected p53 and p38 as well as JNK expressions and the activation and translocation of Bax and cytochrome C. Flow cytometer (FCM) was used to detect the mitochondrial membrane potential and cell apoptosis.* Results. *After separation and culture, 95% of cells showed positive cTnI expression, which indicated that mouse primary cardiomyocytes were successfully identified in our research. DOX activated p53 and MAPK signal pathways in a time-dependent manner, which were inactivated by being cotreated with SYKT, PFT-*α*, or SB, respectively. DOX significantly decreased Bax and increased cytochrome c expressions in the cytoplasm, whereas Bax was upregulated and cytochrome c was downregulated in the mitochondria, which were reversed by SYKT treatment. Besides, DOX reduced mitochondria membrane potential (MMP) in cardiomyocytes compared to the control group; SYKT recovered its MMP and attenuated DOX-induced cardiomyocyte injury. Of note, DOX increased the expression levels of cleaved caspase-3 as well as poly ADP-ribose polymerase (PARP) and promoted cell apoptosis, which were also reversed by SYKT treatment.* Discussion and Conclusions. *Our results indicated that SYKT alleviated DOX-induced cardiotoxicity by inhibiting p53 and MAPK signal pathways' activation-mediated apoptosis, and it might serve as a potential therapeutic agent for DOX-induced cardiotoxicity.

## 1. Introduction

Doxorubicin (DOX) has been widely used as a chemotherapeutic agent for various cancers' treatment because of its high therapeutic efficacy and broad-spectrum effects; however, DOX-induced acute and chronic irreversible cardiotoxicities for patients limited its applications to a large extent [1]. Recent studies have reported that DOX promoted reactive oxygen species (ROS) production in cells, which induced cell intoxication and played an important role in DOX induced cardiotoxicity [2, 3], but the mechanisms are still not fully delineated. High levels of ROS have been reported to induce apoptosis and necroptosis in human pancreatic cancer cells [4] and promoted cell death in HepG2 cells [5]. Notably, MAPK and p53 signal pathways played important roles in this process; high levels of ROS promoted cell autophagy and apoptosis by synergistically activated MAPK [6, 7] and p53 [8, 9] signal pathways. Therefore, ROS might induce cell intoxication by activating MAPK and p53 signal pathways, which promoted cell autophagy and apoptosis. Besides, our preliminary experiments also found that DOX induced cell death and cardiotoxicity in a ROS-production-dependent manner [10], which was in accordance with previous studies. The studies above indicated that eliminating ROS might be an ideal method to alleviate DOX-induced cell intoxication.

Antioxidants were effective for ROS elimination [11]; based on the previous studies, we speculated that antioxidants might alleviate DOX-induced cardiotoxicities by targeting ROS productions. Sanyang Xuedai mixture (SYKT) is a natural medicine and an antioxidant originating from an ancient prescription of the Dai nationality in southwest China [12], which comprised eight primary ingredients:* Daemonorops draco, Panax notoginseng, Scoparia dulcis, Aralia cordata, Alpinia officinarum, Dioscorea opposita, Wolfiporia extensa, *and* Amomum villosum [10]*. SYKT has been verified as a therapeutic agent for DOX-induced cardiotoxicity without impairing its antitumor efficacy by inhibiting ROS-mediated apoptosis [10]. Therefore, it is reasonable to speculate that SYKT might alleviate DOX-induced cell intoxication by eliminating oxidative stresses. However, the mechanisms of this process are still unclear, since MAPK and p53 signal pathways' activation has been reported to be involved in ROS-mediated apoptosis; we speculated that SYKT might attenuate DOX-induced cardiotoxicities by eliminating ROS-mediated p53 and MAPK signal pathways' activation-induced cell apoptosis. Our study will help to determine the mechanisms of SYKT in reducing DOX-induced cardiotoxicity and shed light on novel therapy for the serious cardiac dysfunction induced by DOX in cancer treatment.

## 2. Materials and Methods

### 2.1. Materials

DOX was purchased from Shenzhen Main Luck Pharmaceuticals Inc. (Shenzhen, China) and dissolved in PBS. SYKT (1.02 g/ml) was bought from Great Tao Pharmaceutical Co., Ltd. (Yunnan, China). The JNK, p38, and p53 antibodies were generated by Cell Signaling Technology (Boston, USA). The p53 inhibitor Pifithrin-*α* (PFT-*α*) was from Calbiochem (Germany). p38/MAPK inhibitor SB203583 (SB), JNK inhibitor SP600125 (SP), and Rhodamine l23 were from Sigma-Aldrich Chemical Company (St. Louis, MO, USA). The cardiac troponin I (cTnI), Bax, cytochrome c, caspase-3, and poly-ADP ribose polymerase (PARP) antibodies and various secondary antibodies were produced by Santa Cruz Biotechnology (Santa Cruz, USA).

### 2.2. Animals

13 of inbred C57BL/6J mice (age: 8‐9 weeks; weight 20-25 g) were kindly offered by Dashuo Laboratory Animal Technology (Chengdu, China). The mice were housed in well-ventilated cages (5 mice per cage) under standard conditions. The care of the animals involved in the experiments and procedures was conducted in conformity with the National Institutes of Health (NIH) guidelines (NIH Pub. No. 85-23, revised 1996) and approved by the Animal Care and Use Committee of Kunming Medical College (Kunming, China).

### 2.3. Identification, Separation, and Culture of Mouse Primary Cardiomyocytes

C57BL/6J mice (13 animals were used in this research) were euthanized by neck breaking in 1-4 days after birth; mice hearts were acquired and the ventricular muscle tissues were collected after dissection; the separated tissues were digested with 0.2% trypsin and acquired single cardiomyocyte suspensions. Cells were cultured in 37°C 5% CO_2_ conditions, using Dulbecco's modified Eagle's medium (DMEM) containing 10% fetal bovine serum (FBS), 100 U/mL of penicillin, and 100 *μ*g/mL of streptomycin. The specific marker of cardiomyocytes was cTnI [13]. Purity of cardiomyocyte was evaluated by cTnI immunofluorescent staining. Purity above 95% was regarded as successfully separating cardiomyocytes.

### 2.4. In Vitro DOX and SYKT Treatments

After cardiomyocyte confluence reached above 95%, cells were cocultured with DOX (1uM) alone, SYKT (30mg/ml) alone, or DOX (1uM) plus SYKT (30mg/ml), respectively, for 4 days (based on our preliminary experiments, data not shown). PFT-*α* was set as a positive control for SYKT [14] to detect p53 expression level. SB203580 was used as a positive control for SYKT to detect p38 expression level [15]. SP600125 was used as a positive control for SYKT [16] to detect JNK expression level. Each assay has 3 repetitions.

### 2.5. Western Blot

Total protein (50 mg) was separated by 10% SDS-PAGE, followed by selecting the targeted proteins and transferring them to polyvinylidene fluoride (PVDF) membranes. 5% nonfat dry milk was used to block the membranes at room temperature for 2h, followed by incubation with anti-p53 (1:1000 dilution), anti-p38 (1:1000 dilution), anti-p-JNK (1:1000 dilution), anti-PARP (1:1000 dilution), anti-Bax (1:1000 dilution), anti-caspase-3, and anti-cleaved-caspase-3 (1:100 dilution) primary antibodies, respectively, at 4°C overnight. The membranes were washed with Tris-buffered saline with Tween 20 (TBST; 50 mmol/L Tris-HCl, pH 7.6, 150 mmol/L NaCl, and 0.1% Tween 20) for 30 min and incubated with a horseradish peroxidase- (HRP-) conjugated secondary antibody (1:2000 dilution) for 2 h at room temperature. HRP substrate 3,3'-diaminobenzidine tetrahydrochloride (DAB) system was used to evaluate protein expression levels (Bangalore Genei, India). Each assay has 3 repetitions.

### 2.6. Mitochondrial Membrane Potential (MMP) Detection

Mitochondrial cationic fluorescent dye Rhodamine 123 was used to detect MMP, and the uptake of Rhodamine 123 was employed to evaluate MMP levels [17]. After staining, flow cytometry (FCM) or confocal laser scanning microscopy (CLSM) was used to detect signal intensity under 488 nm (CLSM) or 530 nm (FCM) excitation light wavelength, which reflected MMP changes in cells. FlowJo software was used to analyze the FCM data. The mean fluorescence intensity of control (cont.) group was set as 100%, other groups were normalized according to cont. (% cont. group). Image-Pro Plus 6.0 software was employed to analyze the CLSM data. Each microscopic field (containing at least 200 cells) was photographed and randomly selected for the further analysis. Each assay has 3 repetitions.

### 2.7. Cardiomyocyte Apoptosis Analyzed by FCM

The cardiomyocyte samples mentioned above were collected, washed with PBS, centrifuged at 800 ×g for 6 min, suspended in ice-cold 70% ethanol/PBS, centrifuged at 800 ×g for another 6min, and suspended with PBS. Cells were cocultured with propidium iodide (PI) and FITC-labeled Annexin V for 30 min at 37°C. Cells were washed with PBS; FACSCalibur flow cytometer was used to analyze cell apoptosis (Becton Dickinson, Mountain View, CA, USA). CellQuest software was used to analyze the data. Each assay has 3 repetitions.

### 2.8. Statistical Analyses

All the data collected in our experiments were shown as the mean ± standard deviation (SD), and the data were analyzed by SPSS 13.0 statistical software with one-way analysis of variance (ANOVA) for multiple groups and Student's* t*-test for two groups.* P*<0.05 means statistical significance.

## 3. Results

### 3.1. Culture and Identification of Mouse Primary Cardiomyocytes

**Figure 1:**
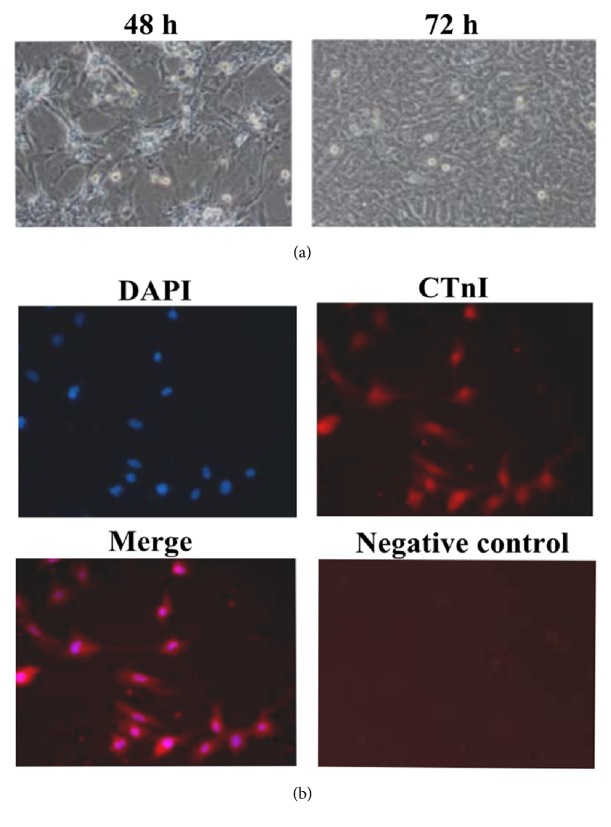
Culture and identification of mouse primary cardiomyocytes. (a) After 48 h of incubation, adherent cardiomyocytes accounted for approximately 90% of the cells. The cells exhibited various morphologies, such as spindle and polygonal shapes. After 72 h of incubation, the cells fused together (10×). (b) Cardiomyocytes identification using cTnI immunofluorescent staining (10×).

Observation for cell morphologies under a light microscope showed that adherent cardiomyocytes accounted for approximately 90% after 48 h of culture. The cell morphologies exhibited spindle and polygonal shapes. The cells had extended pseudopodia and intertwined to form a radial and concentric circular cell population. The majority of the cardiomyocytes exhibited rhythmic and almost synchronized contractive pulsation. The pulsation frequency was primarily 60-100 times/min. After culture for 72 h, the cardiomyocytes had fused together (Figure 1(a)).

Cells were washed with PBS twice to remove dead cells. The cardiomyocytes were identified using cTnI immunofluorescent staining and observed under an inverted fluorescence microscope. The results showed that more than 95% of the cells showed red fluorescence in the cytoplasm (positive CTnI expression) and blue fluorescence in the cell nuclei (Figure 1(b)), which indicated that we have successfully isolated cardiomyocytes from mice heart.

### 3.2. p53 Signal Pathway Was Inactivated by SYKT in DOX-Induced Cell Intoxication

**Figure 2:**
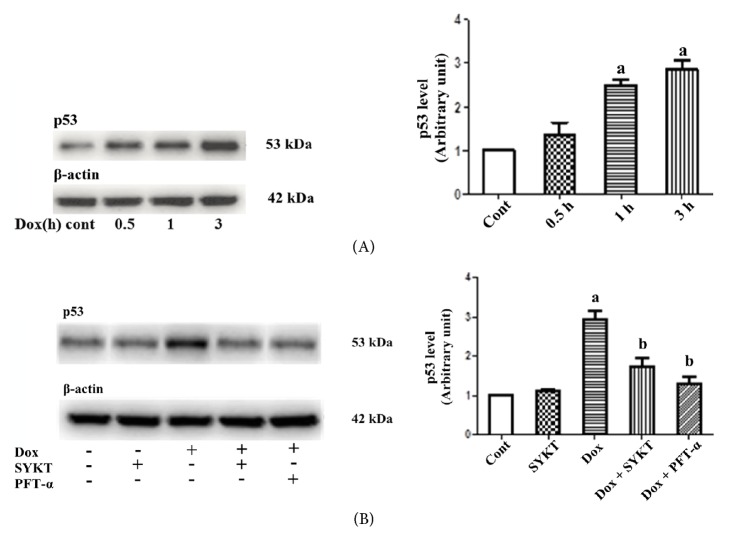
SYKT inhibits DOX-induced p53 activation. (A) DOX induces p53 expression in a time-dependent manner. Cardiomyocytes were treated with DOX for the indicated periods, and the p53 levels were analyzed by immunoblotting. *β*-Actin served as a loading control. (B) Effects of DOX (1 *μ*M for 3 h), SYKT (30 mg/ml), and PFT-*α* (40 *μ*M) on p53 expression. ^a^*p* < 0.05 versus control; ^b^*p* < 0.05 versus DOX;* n* = 6.

Mouse primary cardiomyocytes were treated with DOX and collected at different points (0.5, 1, and 3 h) according to our preliminary results (data not shown). The results showed that p53 signal pathway was activated in a time-dependent manner; its expression reached the highest level at 3 h (Figure 2(A)). PFT-*α* is a specific inhibitor of p53 activation and is used as a positive control for SYKT in this study. After pretreatment of the cardiomyocytes in the combination group with either 30 mg/ml of SYKT or 40 *μ*M PFT-*α* for 2 h, cells were incubated with 1 *μ*M DOX for another 3 h. The level of activated p53 in the cardiomyocytes was evaluated. The level of activated p53 in the cardiomyocytes in the DOX single drug group was significantly increased (^a^*p *< 0.05), whereas the level in the cardiomyocytes pretreated with SYKT or PFT-*α* was significantly decreased (Figure 2(B)). These results suggested that SYKT attenuated DOX-induced cardiomyocyte injury through the inhibition of p53 activation (^b^*p *< 0.05).

### 3.3. SYKT Inhibited MAPK Signal Pathway Activation in DOX-Induced Cardiotoxicity

**Figure 3:**
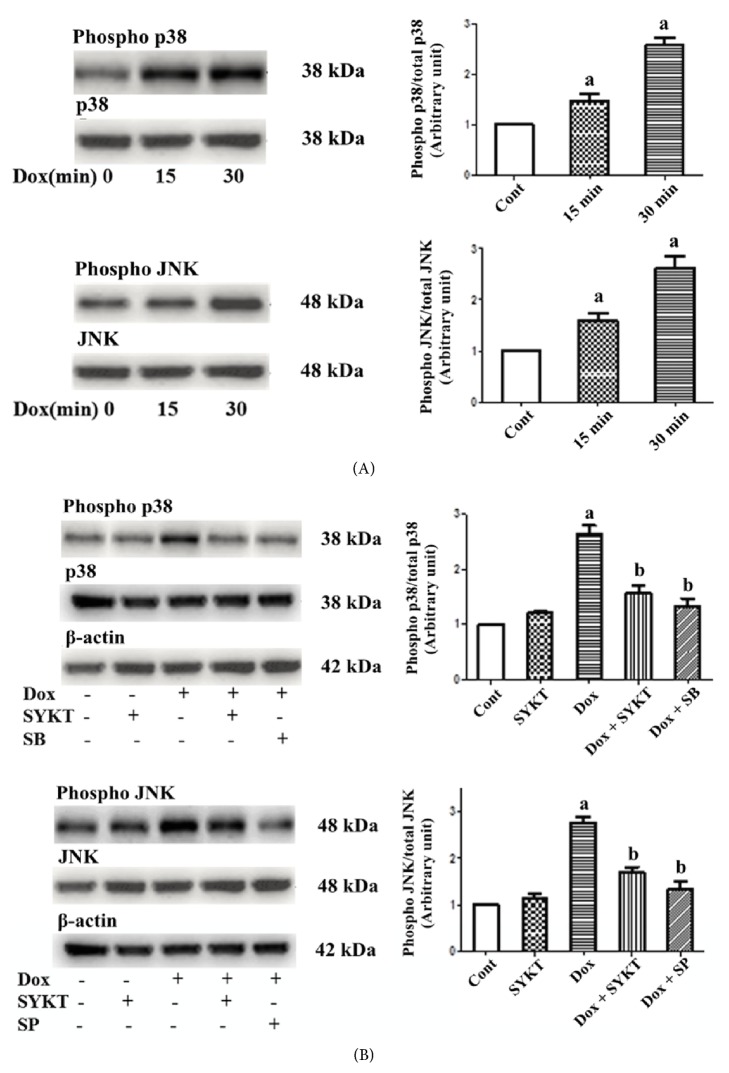
SYKT inhibits DOX-induced MAPK activation. (A) DOX induces p38/JNK MAPK activation in a time-dependent manner. (B) DOX-mediated p38/JNK MAPK activation is inhibited by SB 203580/SP600125 and by SYKT. The cardiomyocytes were treated with SB 203580/SP600125 prior to DOX (1 *μ*M for 30 min) addition. *β*-Actin was used as an internal control. ^a^*p* < 0.05 versus control; ^b^*p* < 0.05 versus DOX;* n* = 6.

Mouse primary cardiomyocytes were treated with 1 *μ*M DOX. Cardiomyocytes were collected at different time points (0, 15, and 30 min) according to our preliminary results (data not shown), and the activated p38 and JNK expression levels were detected by western blotting. The expression levels of these two proteins differed at the different time points. The activated p38 and JNK expression levels gradually increased from 0 to 30 min and reached the highest levels at 30 min (Figure 3(A)).

SB203580 is a specific inhibitor of p38 phosphorylation, and SP600125 is a specific inhibitor of JNK phosphorylation; these inhibitors were used as the positive controls for SYKT in this study. Cardiomyocytes in the combination group were pretreated with 30 mg/ml of SYKT, 20 *μ*M SB, or 10 *μ*M SP for 2 h, followed by incubation with 1 *μ*M DOX for another 30 min. The activated p38 and JNK levels were measured in the cardiomyocytes. The activation levels of these two proteins in the cardiomyocytes in the DOX single drug group were significantly increased (^a^*p *< 0.05), whereas the activation levels in the cardiomyocytes pretreated with SYKT or the inhibitors were significantly decreased (Figure 3(B)). These results suggested that SYKT attenuated DOX-induced cardiomyocyte injury through inhibition of the activation of MAPK-associated proteins (^b^*p* < 0.05).

### 3.4. SYKT's Effects on Proapoptotic Signal Activation

**Figure 4:**
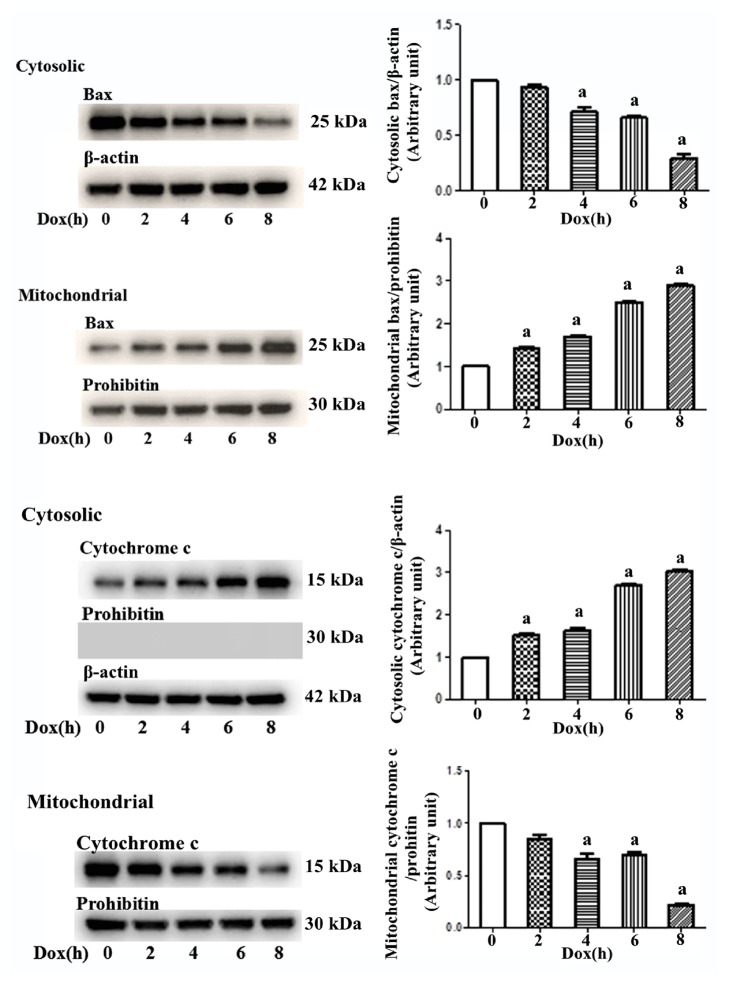
Influence of DOX on cytoplasmic and mitochondrial Bax and cytochrome c expression is time-dependent. Mouse primary cardiomyocytes were treated with 1 *μ*M DOX for 1-8 h. Over time, cytoplasmic Bax expression gradually decreased and cytochrome c expression gradually increased, whereas mitochondrial Bax expression gradually increased and cytochrome c expression gradually decreased. *β*-Actin was used as the internal control, and Prohibitin was used as a mitochondrial marker.

Mouse primary cardiomyocytes were treated with 1 *μ*M DOX, and cells were collected at different time points (0, 2, 4, 6, and 8 h) according to our preliminary results (data not shown). After the cytoplasmic proteins and mitochondrial proteins were isolated, the Bax and cytochrome c expression levels were examined by western blotting. The cytoplasmic and mitochondrial Bax and cytochrome c expression levels differed at the different time points. From 0 h to 8 h, the Bax expression levels gradually decreased and the cytochrome c expression levels gradually increased in the cytoplasm, whereas the Bax expression level gradually increased, and the cytochrome c expression level gradually decreased in the mitochondria (Figure 4).

**Figure 5:**
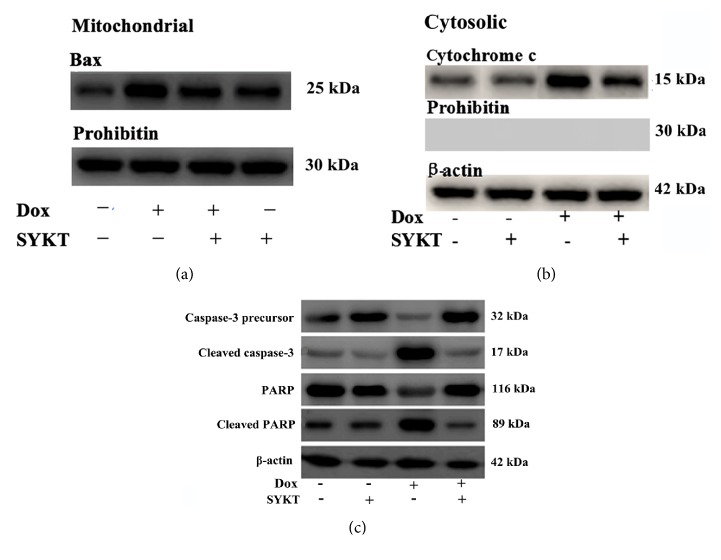
Effects of SYKT on DOX-induced cardiomyocyte apoptosis-related proteins at 8 h after DOX processing. (a) The Bax levels were analyzed in the mitochondrial extracts by immunoblotting. (b) Effects of DOX and SYKT on cytoplasmic cytochrome c release in cardiomyocytes. (c) Western blotting analysis of the effects of DOX, SYKT, and DOX+SYKT on caspase-3 activation and PARP cleavage in cardiomyocytes. *β*-Actin was used as the internal control, and Prohibitin was used as a mitochondrial marker.

Cardiomyocytes in the combination group were pretreated with 30 mg/ml of SYKT for 2 h, followed by incubation with 1 *μ*M DOX for another 8 h; the time points were selected according to our preliminary results (data not shown). The mitochondrial Bax, cytoplasmic cytochrome c, and cellular caspase-3 expression levels and the activated fragment of cleaved PARP were examined in the cardiomyocytes. The expression levels of the above proteins in the cardiomyocytes in the DOX single drug group all significantly increased, whereas this trend of an increase in protein expression was significantly inhibited after combined treatment with SYKT (Figure 5). These results indicate that SYKT is a potent prosurvival factor that inhibits DOX-induced cardiomyocyte death by reversing mitochondrial Bax translocation and inhibiting cytochrome c release to the cytosol.

### 3.5. MMP Was Detected in SYKT and DOX-Treated Cardiomyocytes

**Figure 6:**
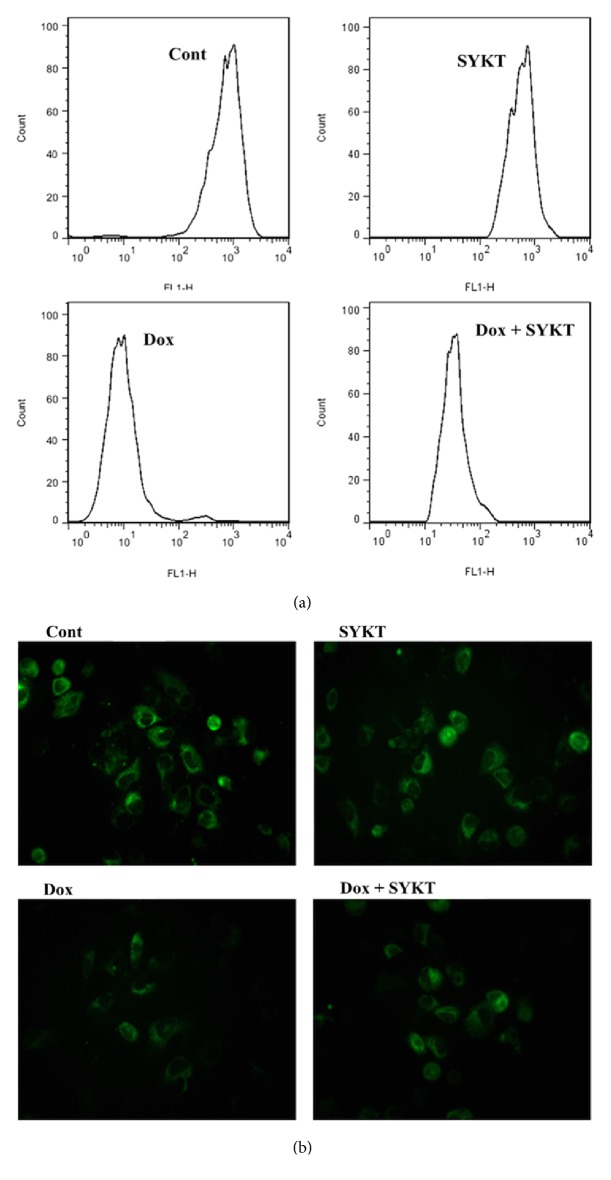
Determination of the MMP of cardiomyocytes from each group (*n* = 6 rats/group) at 8 h after DOX processing. (a) Histograms of the MMP of cardiomyocytes detected by Rhodamine 123 staining by FCM. (b) Photos of Rhodamine 123 staining of MMP in all groups by CLSM (100×).

FCM detection of Rhodamine l23 staining showed that the peak of the histogram of the cardiomyocytes in the DOX single drug group significantly shifted to the left, whereas the peak of the SYKT/DOX combination group significantly shifted to the right. These results suggested that SYKT attenuated the DOX-induced reduction in the MMP in the cardiomyocytes (Figure 6(a)). Statistical analyses were performed on the relative mean fluorescence intensities in the cardiomyocytes in all groups. Compared with the MMP in the cont. group (100%), the relative mean fluorescence intensity in the cardiomyocytes in the DOX single drug group was significantly decreased (39.4 ± 2.9%) (^a^*p* < 0.05), whereas the relative mean fluorescence intensity in the cardiomyocytes after combination treatment with SYKT increased to 69.8 ± 5.5% (^b^*p* < 0.05) (Table 1).

CLSM technology using Rhodamine 123 staining showed that the green fluorescence visible in the images of cardiomyocytes in the DOX single drug group was significantly decreased compared with the cont. group, whereas the expression of green fluorescence in the SYKT/DOX combination group was significantly increased (Figure 6(b)). Statistical analysis of the relative mean fluorescence intensities in the cardiomyocytes in all groups was performed. The relative mean fluorescence intensity in the cardiomyocytes in the DOX single drug group was 25.1 ± 2.5%, which was significantly decreased compared with the MMP (100%) in the cont. group (^a^*p* < 0.05). After pretreatment with combined SYKT, the relative mean fluorescence intensity increased to 58.4 ± 6.3% (^b^*p* < 0.05) (Table 1).

The results obtained with the two detection methods suggested that SYKT attenuated cardiomyocyte injury through inhibition of the DOX-induced reduction in the MMP.

### 3.6. SYKT Attenuated DOX-Induced Cardiomyocyte Apoptosis

**Figure 7:**
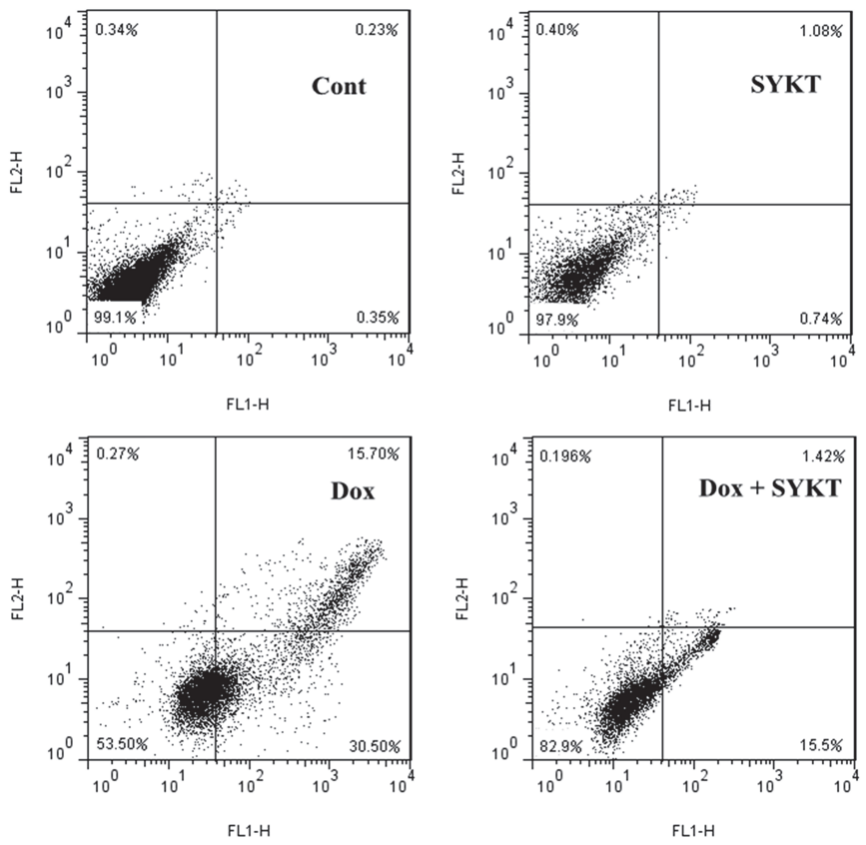
Percent distribution of apoptotic and necrotic cells from each group (*n* = 6 rats/group) at 8 h after DOX processing. The cell distribution was analyzed using Annexin V binding and PI uptake. The FITC and PI fluorescence was measured using a FCM with FL-1 and FL-2 filters, respectively.

For the more accurate evaluation of the impact of DOX and SYKT on cell viability involved in the process of cardiomyocytes dysfunction and death, the cells from all experimental groups were assessed by FCM. The data (Figure 7) revealed that the DOX-exposed cardiomyocytes showed maximum Annexin V-FITC-binding (30.50%) but very little PI staining (0.27%) compared to the control, which indicated that DOX significantly increased cell apoptosis rates. In the groups of cardiomyocytes treated with SYKT, cell apoptosis rates were significantly decreased (1.42%) compared to the DOX-treated group (15.5%), which indicated that SYKT protected the cardiomyocytes from DOX-induced apoptosis.

## 4. Discussion

DOX is an antibiotic and is widely used as an antitumor drug for various cancers' treatment including hematopoietic malignancies [18], malignant lymphoma [19], breast cancer [20], non-small cell lung cancer [21], and mesenchymal tumor [22]. However, besides its therapeutic effects, DOX induced severe cardiotoxicity by increasing ROS-mediated apoptosis and limited its application in clinical practices [23, 24], which indicated that targeting ROS might be an effective method to alleviate DOX-induced side effects in cancer treatment. SYKT was an antioxidant and our preliminary experiment has proven that SYKT helped to alleviate DOX-induced cardiotoxicity by inhibiting ROS-mediated cell apoptosis [10], but the mechanisms are still unclear.

In this study, we first identified and separated cardiomyocytes from mice's ventricular muscle tissues, and above 95% of the total cells have been verified as cardiomyocytes, which indicated that we have successfully segregated mice cardiomyocytes. Cell apoptosis was detected and the results showed that DOX increased ROS-mediated cardiomyocytes apoptosis in a time-dependent manner, which was reversed by coculturing with SYKT. Our further results also showed that the expression levels of apoptosis-associated proteins including the cleaved caspase-3 and PARP were elevated; interestingly, MMP was reduced by DOX treatment; as a result, cytochrome c was released from mitochondria into cytoplasm, which verified that DOX induced ROS-mediated cell apoptosis, and these results were in accordance with the previous study [25]; by coculturing SYKT and DOX with cells, we found that SYKT reversed these phenomena. The results above suggested that SYKT alleviated DOX-induced ROS-mediated cell apoptosis.

ROS has been reported to induce cell apoptosis by activating p53 and MAPK signal pathways [8, 9, 26], but it is still unclear whether ROS-mediated activation of p53 and MAPK signal pathways has participated in SYKT-mediated alleviation of DOX-induced cardiotoxicity. Therefore, we hypothesized that SYKT inhibited DOX-induced cell apoptosis and cardiotoxicity by eliminating ROS and inhibited ROS-mediated p53 and MAPK signal pathways' activation, which helped inhibit cell apoptosis. To verify our speculation, we detected p53 and MAPK signal pathways' activation in SYKT and DOX treatment groups; the results showed that DOX increased p53 expression levels; SYKT or the p53 inhibitor PFT-*α* significantly inhibited p53 expression and activation, which indicated that SYKT decreased cell apoptosis by inhibiting p53 signal pathway. In addition, previous studies suggested that oxidative stress activated MAPK family proteins including p38 and JNK MAPKs [27], which have been proven to play critical roles in the DOX-induced cell death pathways [28]. We also found that DOX promoted p38 and JNK MAPKs phosphorylation in cardiomyocytes in a time-dependent manner, and these effects were significantly reversed by adding MAPK signal pathway inhibitors (SB 203580 and SP600125) and SYKT. The results above suggested that p53 and MAPK signal pathways were involved and played important roles in the process of SYKT-alleviated DOX-induced ROS-mediated cell apoptosis.

Taken together, our results indicated that SYKT alleviated DOX-induced cardiotoxicity by inhibiting ROS-mediated p53 and MAPK signal pathways' activation-induced cell apoptosis.

## 5. Conclusion

In this study, we found that SYKT decreased DOX-induced cardiomyocyte apoptosis by inhibiting ROS-mediated p53 and p38-JNK signal pathways' activation-induced cell apoptosis, and SYKT might serve as a novel and potential therapeutic agent for DOX-induced cardiotoxicity.

## Figures and Tables

**Table 1:** Detection of the MMP in cardiomyocytes in all groups using Rhodamine 123 staining.

Group	Relative mean fluorescence intensity (% cont. group)	
	FCM	CLSM
Cont. group	100	100
SYKT single drug group	85.0 ± 4.5	79.2 ± 3.7
DOX single drug group	39.4 ± 2.9^a^	25.1 ± 2.5^a^
SYKT/DOX group	69.8 ± 5.5^b^	58.4 ± 6.3^b^

MMP: mitochondrial membrane potential; FCM: flow cytometry; CLSM: confocal laser scanning microscopy.

The data are expressed as $\bar{x}\pm s$.

^*a*^ *p* < 0.05 versus control; ^*b*^*p* < 0.05 versus DOX; *n* = 6.
